# Forecasting emergence of COVID-19 variants of concern

**DOI:** 10.1371/journal.pone.0264198

**Published:** 2022-02-24

**Authors:** James Kyle Miller, Kimberly Elenberg, Artur Dubrawski

**Affiliations:** 1 Auton Systems LLC, Pittsburgh, PA, United States of America; 2 United States Department of Defense Covid Task Force, Washington, DC, United States of America; Boston College, UNITED STATES

## Abstract

We consider whether one can forecast the emergence of variants of concern in the SARS-CoV-2 outbreak and similar pandemics. We explore methods of population genetics and identify key relevant principles in both deterministic and stochastic models of spread of infectious disease. Finally, we demonstrate that fitness variation, defined as a trait for which an increase in its value is associated with an increase in net Darwinian fitness if the value of other traits are held constant, is a strong indicator of imminent transition in the viral population.

## Introduction

RNA viruses such as SARS-CoV-2 have high mutation rates, which allows them to rapidly adapt to changing environments. Fortunately, most mutations are deleterious, and high deleterious mutation loads can be limiting even to the point of error catastrophe [1], extinction as a result of excessive mutations, in the most extreme cases. Even so, antigenic escape is a significant concern with rapidly mutating viruses, since such escape could challenge pandemic control efforts. Some RNA viruses such as influenza A/H3N2 exhibit frequent antigenic escape, with the most recent common ancestor rarely more than 3-5 years in the past [2]. Other viruses such as measles, mumps, or HCV may take decades or even centuries to develop significant antigenic mutations [3]. When, antigenic escape does occur, it can trigger an escalation of new infections as the novel variant is able to reinfect previously immune hosts. Other effects of such mutations such as increased transmission rates, when they arise, result in the novel strain quickly dominating the (antigenically similar) viral population with a speed that is dictated by the magnitude of the relative selective advantage.

Therefore, monitoring for the emergence of antigenic escape or increased transmission rate within the SARS-CoV-2 population is a capability that is fundamentally important for controlling the pandemic. Genetic sequencing of isolates is the primary monitoring framework, but it requires deciding what proportion of isolates will be sampled from each subpopulation being monitored. Toward that end, we explore to what degree and how the tools of population genetics can inform monitoring processes. At the heart of this problem lies the key question: Where and when will the next variant of concern arise?

On its face, this question seems impossible to answer. Indeed, it is often presumed that evolution, being a complex and random process, is by its nature unpredictable [4, 5]. Yet, it has been demonstrated that fitness can be forecast for short time horizons [5]. Further, viral populations are subject to dynamical processes that govern host infection and transmission. These dynamics, often over-simplified into compartmental Susceptible-Infected-Resistant (SIR) models [6, 7] and other similar models, can be used to understand the direction of selective pressure. Thus, there is reason to hope that while one may not be able to know where the next variant of concern will arise, one could make educated estimations. Aligning genetic monitoring activities with the probability distribution of variant emergence will improve efficiency and increase chances of identifying novel variants early.

## Related work

[8] ran a number of simulations and then trained and evaluated machine learning models designed to forecast the rise of novel antigenic types. The authors of this study were researching whether having identified a novel antigenic type, one can predict whether it will rise to a critical relative frequency of infections. This differs from the present work, in which we consider whether we can predict this event without having to first identify a novel strain at all. That is, we attempt to answer the question: will a novel type arise?

### Multi-strain dynamics

Much research focusing on the emergence of novel strains assume the pre-existence of these strains at very low levels [9–12]. In this setting, models of multi-strain dynamics can predict where and when these strains may be detected (i.e., reach sufficient levels) as a function of relative fitness. Similar models can describe the fixation of strains that have higher transmission rates, longer recovery times, or other competitive advantages. The models can also include strain-to-strain mutation rates, which serve as additional source/sink terms.

**Fig 1:**
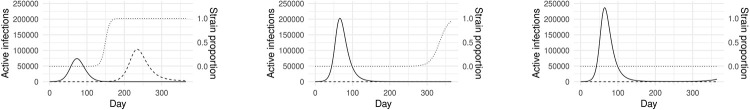
Example simulation results from a two-strain SIRS model with a vaccinated population, for different vaccination rates. The solid line shows the number of active infections for Strain 1. The dashed line shows the number of active infections for Strain 2. The dotted line shows the proportion of the two strains. Vaccine resistant strain initially present at rate of 10^−6^. (**a**) 50% population vaccinated in 100 days. (**b**) 10% population vaccinated in 100 days. (**c**) No vaccination.

Fig 1 illustrates how this works with a two-strain SIRS model, detailed in Appendix A. Like an SIR model, SIRS models describe the rates of change of the number of individuals who are susceptible, infected, or resistant. However, unlike an SIR model, SIRS models include loss of immunity over time, thus resistant individuals can become susceptible. The solid line shows the number of active infections for Strain 1 (wild-type). The dashed line shows the number of active infections for Strain 2 (mutant). The dotted line shows the proportion of infections caused by the two strains. In this example scenario, Strain 2 has escaped the immune response elicited by the vaccine (otherwise, Strains 1 and 2 are equivalent and infection by either confers immunity to the other). Aggressive vaccination of the population leads to the rapid dominance by Strain 2 and includes an overall reduction in peak infections. A lower vaccine rate delays the dominance of Strain 2, while no vaccination results in Strain 2 remaining in obscurity. Here, the initial proportion of Strain 2 is 10^−6^. Note that under this simple model, vaccination confers a competitive advantage to Strain 2 and its dominance is inevitable. Different choices of parameter values only change the timing of this outcome.

A real challenge with these models is that the time at which the mutant strain reaches detectable levels within the population is very sensitive to its initial population, which cannot be experimentally measured. If one does not assume pre-existence, then one must describe the dynamics by which novel strains come into being, i.e. mutation.

### A neutral model of genetic diversity

The simplest form of mutation models are models of neutral mutation. Here, it is presumed that mutations have no effect on fitness. The aim then, is to describe the level of genetic diversity in a population over time. [13] adapt a model of mean number of pairwise differences between sequences under varying population size first proposed by [14], to estimate genetic diversity over time during a viral outbreak. This simple model is given as

$$\begin{array}{c} \frac{d}{dt}\pi =2U-2\pi \left(t\right)\frac{\tau +\mu }{I(t)} \end{array}$$ (1)

where *π* is the mean number of pairwise differences, *U* is the mutation rate, *τ* is the recovery rate, *μ* is the host population birth rate, and *I* is the number of infected individuals. This model can use the retrospective data and forecast numbers of active cases (currently infected individuals) obtained from any relevant case forecasting model, to provide an estimate of within-strain genetic diversity.

### Phylodynamic models

A significant challenge with modeling genetic mutation is that one must also describe how genotype relates to phenotype. Much is known about the SARS-COV-2 virus genome [15] including site specific mutation rates [16]. Yet, to model the genotype-phenotype relationship may require understanding how mutations affect protein folding and resulting protein-protein interactions. Computational approaches for exploring these factors exist [17], but are likely too computationally expensive to comprehensively characterize the genetic landscape.

A common alternative approach is to describe the change of fitness directly [5, 18, 19]. Fitness dynamics can be described using the following integrodifference equation

$$\begin{array}{c} \frac{\partial }{\partial t}u=\left(x-\bar{x}\right)u+\mu (u*g-u), \end{array}$$ (2)

where *x* is a scalar quantity measuring fitness, *u*(*x*, *t*) is proportion of the population with fitness *x* at time *t*, *u* * *g* = ∫_Ω_ *u*(*x* − *τ*, *t*)*g*(*τ*, *t*)*dτ*, *μ* is the mutation rate, and $\bar{x}=\int_{-\Omega }xu\;dx$ is the average population fitness. The shape and properties of the mutation likelihood *g* controls the properties of the fitness trajectories [18]; either a train of well separated beneficial mutations rising to fixation or a traveling wave moving in the direction of increasing fitness.

Compartmental models can be combined with fitness evolution relatively easily, under the assumption of a single antigenic cluster. For example, an SIR model with mutation is given below. However, modeling multiple antigenic clusters requires model extensions [20] which are not easily handled by deterministic frameworks. Instead, stochastic [21] and deterministic-stochastic hybrid [2] methods are used.

### Empirical data

Resources such as GISAID [22] and Nextstrain [23] have made phylogenetic analysis of tens of thousands of SARS-CoV-2 isolates available. The observed phylogeny show contemporary clades displacing earlier ones. The turn over in clades shows ongoing fitness evolution. Some clades including 20H/501Y.V2, 20C/S:452R, and 20J/501Y.V3 show growing antigenic distances.

## Methods & results

An important design choice in modeling variant emergence is whether to consider antigenic mutation. In the absence of antigenic mutation, the models simplify and are more readily treated with deterministic systems. Including antigenic mutation is most readily handled using agent based stochastic models.

### Viral phylodynamics in the absence of antigenic mutation

In the absence of antigenic mutation, the phylodynamics of a viral population can be captured by a small modification to common compartmental models. By considering infections *I*(*β*, *γ*, *t*) to be a function of both time *t* and phenotype *β*, *γ* one arrives at a familiar compartmental formulation in the population totals and one additional equation describing the proportion of infections u=II¯.

$$\frac{d}{dt}S=-\bar{\beta }\bar{I}\frac{S}{S_{0}},$$ (3)

$$\frac{d}{dt}\bar{I}=\bar{\beta }\bar{I}\frac{S}{S_{0}}-\bar{\gamma }\bar{I},$$ (4)

$$\frac{\partial }{\partial t}u=(\beta \frac{S}{S_{0}}-\gamma -\bar{\beta }\frac{S}{S_{0}}+\bar{\gamma })u+\mu (u*g-u).$$ (5)

Here, *S* is the size of susceptible population, $\bar{I}=\int_{\Omega }I\;d\beta d\gamma$ is the total number of infections. $\bar{\beta }$ and $\bar{\gamma }$ are the population averaged transmission and recovery rates, and *g* is the mutation kernel. A derivation of the above can be found in the Appendix B. Note that (5) describes continuous mutation and naturally re-derives (2) in the infectious disease context. The equation can be modified to describe mutation only at the time of transmission, as in [21], resulting in the last term changing to *μS*[(*βu*) * *g* − *βu*]. Both forms are approximations, allowing one to avoid modeling within host dynamics.

From Eq (5) one can see that fitness is defined by the scalar quantity $\beta \frac{S}{S_{0}}-\gamma$ and that fitness is maximally increased in the direction 〈SS0,−1〉. This model does not explicitly include immune escape. The selective pressure in favor of immune escape can be characterized, however. If a genotype with parameters *β* and *γ* mutates in a way to achieve partial immune escape, parameterized by *δ* ∈ [0, 1], then its instantaneous fitness will be β(δ(S0−S)+S)S0−γ. The fitness gradient then becomes

∇=〈SS0,−1,β(1−SS0)〉, (6)

where the last position represents immune escape. Eq (6) makes it clear that selective pressure changes over the course of an outbreak and can be described in roughly three phases. Initially, susceptible hosts are plentiful and fitness is conferred by both increasing *β* and decreasing *γ* which describes the length of time a host spreads the virus. As host availability decreases selective pressure favors variants that are able to spread longer (lower *γ*), but transmissibility becomes less important. With yet further reduction in available hosts, pressure begins to strongly favor immune escape which would increase the number of available of hosts.

**Fig 2:**
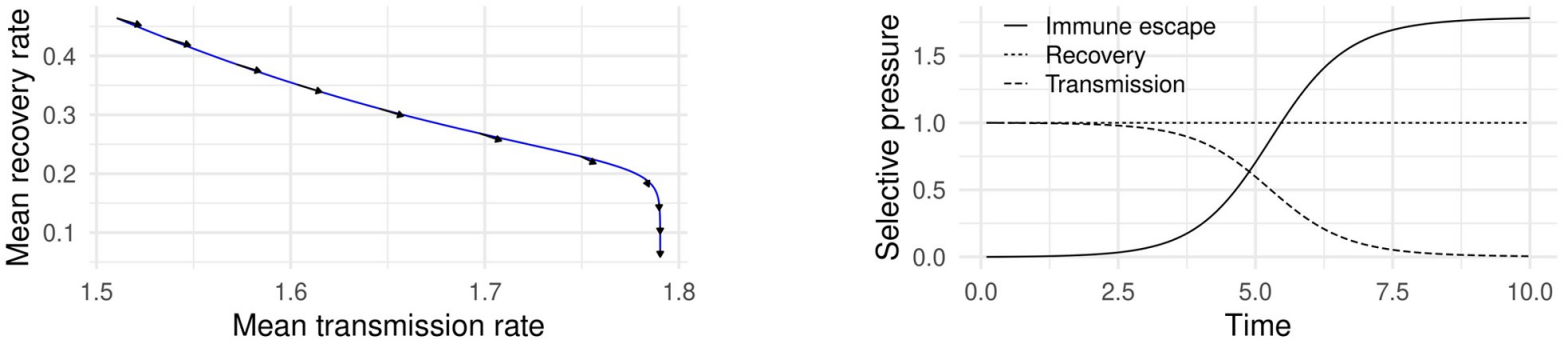
Mean phenotype and selective pressure over the course of a simulated outbreak using (3)–(5). (**a**) $\bar{\beta }$ and $\bar{\gamma }$ over the course of a simulated outbreak. Arrows indicate the direction of 〈SS0,−1〉. (**b**) Magnitude of selective pressure using (6) assuming that no immune escape variant has emerged.

Fig 2 shows simulation results from system (3)–(5) using a Gaussian mutation kernel *g*. Fig 2a shows the mean transmission rate *β* and recovery rate *γ* over the course of a simulated outbreak. The arrows indicate the direction 〈SS0,−1〉 of maximal selective pressure in the (*β*, *γ*) plane. This figure demonstrates that the gradient in (6) agrees with simulation with isotropic mutation kernels and no small-population effects (see [18]). Fig 2b shows the magnitude of selective pressure given by (6) assuming that no immune escape variant has emerged. This figure illustrates the three phases of pressure direction described above.

### Viral phylodynamics in the presence of antigenic mutation

**Fig 3:**
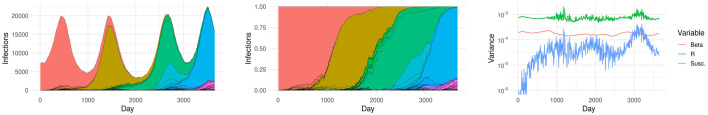
Example simulation representing a 10 yr period. Antigenic types are separated by both line and color, though many colors appear similar. (**a**) Infections by antigenic type. (**b**) Antigenic type proportions. (**c**) Variance of fitness parameters.

Antigenic mutations are simpler to simulate using stochastic agent based models and similar techniques, as compared to deterministic compartmental models. We therefore adapted an existing agent based model for influenza A/H3N2 which includes mutation in both transmission rate and the antigenic space [21]. We sampled key model parameters independently and uniformly over a range of plausible values, with the exception of population size for which log_10_ population size was sampled uniformly. These key parameters, their descriptions, and range of values are given in Table 1. Using default parameters, this model shows spindly phylogeny, with novel antigenic variants rising to fixation approximately every three years. For the purpose of illustration, Fig 3 shows an example simulation representing a year period. Fig 3a shows weekly infections colored by antigenic type. Note that colors for different types may appear similar and the contribution of each type is separated by black lines. Fig 3b shows the proportion of infections caused by each type over time. Fig 3c shows the variance of key fitness measures over time. These are the proportion of the population that is susceptible (susc.), the transmission rate which includes the effects of deleterious mutation load (beta), and the reproduction value (R). Note that there are three periods of transition of dominant antigenic type which correspond to three periods of increased variance in fitness.

**Table 1:** Range of perturbed simulation parameters. Each parameter was sampled uniformly over the indicated range.

Parameter	Description	Range
log_10_(initialNs)	log Population size	[4, 8]
initialPrR	Initial strain resistance	[0, 0.5]
beta	Base transmission rate	[0.3, 0.6]
nu	Recovery rate	[0.15, 0.25]
lambda	Non-antigenic mutation rate (mean number of mutations per transmission event)	[0.05, 0.25]
mutCost	Base transmission rate cost for deleterious non-antigenic mutations	[0.006, 0.01]
epsilon	Probability of beneficial non-antigenic mutation	[0.1, 0.2]
lambdaAntigenic	Antigenic mutation rate (mean number of mutations per transmission event)	[0.00075, 0.001]

**Fig 4:**
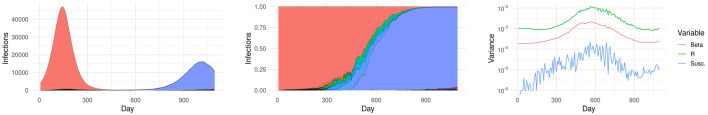
Example sample simulation, showing fixation of a novel variant. Antigenic types are separated by both line and color, though many colors appear similar. (**a**) Infections by antigenic type. (**b**) Antigenic type proportions. (**c**) Variance of fitness parameters.

Similarly to [8], we ran a number of simulations and then trained and evaluated machine learning models designed to forecast the rise of novel antigenic types. We ran 2,000 simulations, each simulating a 3 year period. Fig 4 includes a single example, showing infections by antigenic type, proportion of types, and variance of fitness measures as in Fig 3. The third year in each simulation was discarded, only being used to determine whether an antigenic type would reach 5% relative infection frequency. We refer to antigenic types reaching 5% relative frequency as novel variants. Other types that do not reach this threshold are out competed and not considered novel variants. For each simulation, the weeks between origination and the time at which a novel variant first reaches 5% relative frequency are labeled as ‘positive’, indicating the presence of a latent novel variant. The weeks after a novel variant attains 5% relative frequency are discarded. All other weeks are labeled as ‘negative’.

Simulations in which no antigenic types originate beyond the initial type were discarded, leaving 1,510 simulations. This was done because when no novel antigenic types originate, susceptibility variance is identically zero. Exactly zero variance has two consequences. First, variance becomes incredibly informative resulting in prediction performance significantly higher than reported below (AUC 0.933), since the resulting negative samples fall below any nonzero threshold. Second, determining that the true variance is truly zero in real-world applications is problematic. This issue is further explored in the discussion below.

Each week is then featurized by the current number of infections, cumulative infections, total number of susceptible hosts, population size, and the means and variances of susceptible host proportion (susc.), the transmission rate (beta), and the reproduction value (R). Finally, we append the previous week’s values as additional features. We trained and evaluated a Random Forest using blocked 10 fold cross-validation, wherein weeks from the same simulation where not allowed to be split across multiple folds.

**Fig 5:**
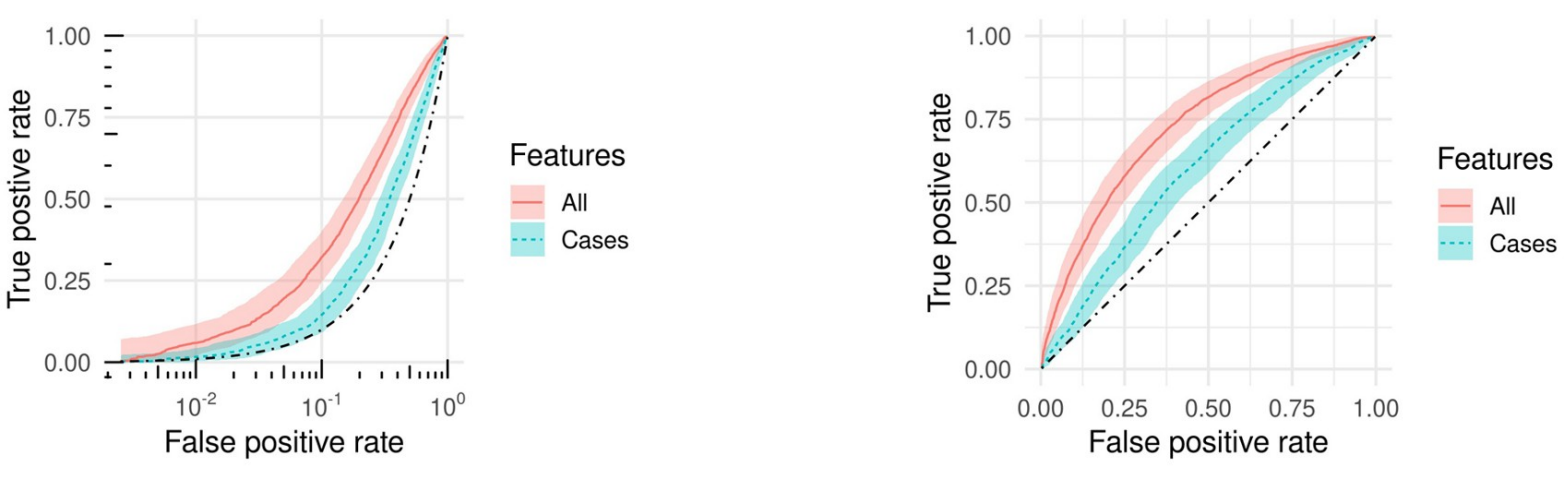
ROC curves of model predicting the emergence of a novel variant. The dotted black line indicates random performance. (**a**) Log scale. (**b**) Linear scale.

Fig 5 shows Reciever Operator Characteristic (ROC) curves for Random Forests trained using all features (red, solid) and using only case count based features (i.e. no fitness features) (blue, dashed). The black dash-dot line indicates the random line. Using all features the model achieves an area under the ROC curve (AUC) of 0.731, while using only case count based features the model achieves an AUC of 0.606. Using only the variance of susceptible host proportion for the current and previous week is sufficient to achieve an AUC of 0.716, indicating that antigenic variation is a leading indicator of novel strain emergence.

## Conclusion

We have demonstrated that systematic properties of population dynamics in infectious diseases can inform the likelihood of the random process of mutation. When mutation is strong, the traveling wave of the fitness distribution tends to move in the direction of the fitness gradient. When mutation is weak, the fitness dynamics are characterized by periodic substitution events [18]. In this case, variation in population fitness is a strong indicator that such an event is imminent. Our simulation results support these conclusions.

In order to apply these findings to the SARS-CoV-2 pandemic, we must be able to measure variation within the viral population. In the absence of this capability, our results indicate that one would suffer a significant decrease in predictive power. As mentioned above, while isolates can increasingly be sequenced and their genetic sequences analyzed, one must be able to link genotype to the relevant aspects of phenotype or fitness. Certain types of mutations, those located in the spike protein for example are more likely to have an impact on fitness, but quantifying that impact may be difficult. Neutralizing monoclonal antibody testing [24] may serve as a suitable proxy. In addition, increased variation in antibody response to collected isolates may well indicated increased antigenic variation. However, more study is required to quantify these relationships.

We expected the gradient in Eq (6) to be much more informative of outcomes of the stochastic agent based simulations. This proved not to be the case. Mutations arise at a rate proportional to the number of transmission events and as the fitness gradient changes, the fate of a mutant may become favorable. While this fitness gradient can inform these changes, the range of parameter values chosen created such large variation in susceptible host proportion, as well as trajectories of the same, that the relationship between susceptible host proportion and probability of a novel variant emerging was overwhelmed. This may be appropriate however, since considerable uncertainty continues to remain concerning the most appropriate models and parameter values to describe the SARS-CoV-2 outbreak. This will likely be the case in future outbreaks as well.

## Appendices

### A Two-strain SIRS model with vaccination

A sample two-strain competitive SIRS model with vaccination. Vaccination is assumed to confer permanent immunity against strain 1 but none against strain 2. Infection by either strain confers temporary immunity to both. Table 2 summarizes model variables, parameters, and parameter values.

$$\frac{d}{dt}S=\mu N-v\sigma \left(S\right)-\beta_{1}SI_{1}-\beta_{2}S\left(I_{2,S}+I_{2,V}\right)-\mu S+\gamma R_{1}+\gamma R_{2,S}$$ (7)

$$\frac{d}{dt}V=v\sigma \left(S\right)-\beta_{2}V\left(I_{2,S}+I_{2,V}\right)-\mu V+\gamma R_{2,V}$$ (8)

$$\frac{d}{dt}I_{1}=\beta_{1}SI_{1}-\left(\tau +\mu \right)I_{1}$$ (9)

$$\frac{d}{dt}I_{2,S}=\beta_{2}S\left(I_{2,S}+I_{2,V}\right)-\left(\tau +\mu \right)I_{2,S}$$ (10)

$$\frac{d}{dt}I_{2,V}=\beta_{2}V\left(I_{2,S}+I_{2,V}\right)-\left(\tau +\mu \right)I_{2,V}$$ (11)

$$\frac{d}{dt}R_{1}=\tau I_{1}-\left(\gamma +\mu \right)R_{1}$$ (12)

$$\frac{d}{dt}R_{2,S}=\tau I_{2,S}-\left(\gamma +\mu \right)R_{2,S}$$ (13)

$$\frac{d}{dt}R_{2,V}=\tau I_{2,V}-\left(\gamma +\mu \right)R_{2,V}$$ (14)

**Table 2:** Two-strain SIRS model variables and parameters used in simulations.

Variable	Description
*S*; *S*(0) = *N* − *I*_1_(0) − *I*_2,*S*_(0)	Susceptible but unvaccinated population
*V*; *V*(0) = 0	Vaccinated and susceptible population
*I*_1_; *I*_1_(0) = 100	Individuals infected by Strain 1
*I*_2,*S*_; *I*_2,*S*_(0) = 10^−4^	Unvaccinated individuals infected by Strain 2
*I*_2,*V*_; *I*_2,*V*_(0) = 0	Vaccinated individuals infected by Strain 2
*R*_1_; *R*_1_(0) = 0	Individuals recovered from Strain 1
*R*_2,*S*_; *R*_2,*S*_(0) = 0	Unvaccinated individuals recovered from Strain 2
*R*_2,*V*_; *R*_2,*V*_(0) = 0	Vaccinated individuals recovered from Strain 2
*N* = 10^6^	Total population size
*μ* = 3 ⋅ 10^−3^	Population birth and death rate
*v* = 5000, 1000, 0	Vaccination rate
*σ*(*S*)	1-Sigmoid function to stabilize system: $\frac{\text{exp}(0.25S-25)}{1+\text{exp}(0.25S-25)}1_{S>0}$
*β*_1_ = 2.5 ⋅ 10^−7^	Transmission rate for Strain 1
*β*_2_ = 2.5 ⋅ 10^−7^	Transmission rate for Strain 2
γ=1365	Loss of immunity rate
τ=110	Recovery rate

### B SIR model with mutation

Let *I*(*β*, *γ*, *t*) be the number of infections by a pathogen with transmission rate *β* and recovery rate *γ* at time *t*. Let *S*(*t*) be the number of susceptible individuals in the population.

The total number of infections is given by $\bar{I}\left(t\right)=\int_{\Omega }I\;dA$. The proportion of infections with a given value of *β* and *γ* is u(β,γ,t)=II¯.

Susceptible individuals are infected at a rate of

$$\begin{array}{c} \frac{d}{dt}S\left(t\right)=\int_{\Omega }-\beta I\left(\beta ,\gamma ,t\right)\frac{S(t)}{S_{0}}\;dA. \end{array}$$ (15)

Similarly, the number of infections changes at a rate of

$$\begin{array}{c} \frac{d}{dt}I\left(\beta ,\gamma ,t\right)=\beta I\left(\beta ,\gamma ,t\right)\frac{S(t)}{S_{0}}-\gamma I\left(\beta ,\gamma ,t\right)+\mu (I(\beta ,\gamma ,t)*g-I(\beta ,\gamma ,t)), \end{array}$$ (16)

where $I*g=\int_{0}^{\infty }\int_{0}^{\infty }I\left(\beta -\tau ,\gamma -\eta ,t\right)g\left(\tau ,\eta ,t\right)\;d\beta d\gamma$ is the convolution of the infected population with the mutation kernel *g*. This assumes that mutation of the pathogen changes the status of entire host. In reality, deleterious mutations within a host will be out-competed and will not fix within the host. This can be addressed by choice of *g*.

Let $\bar{\beta }=\int_{\Omega }\beta u\;dA$ and $\bar{\gamma }=\int_{\Omega }\gamma u\;dA$. Then,

$$\begin{array}{c} \frac{d}{dt}S=\int_{\Omega }-\beta I\frac{S}{S_{0}}\;dA=-\frac{S}{S_{0}}\bar{I}\int_{\Omega }\beta \frac{I}{\bar{I}}\;dA=-\frac{S}{S_{0}}\bar{I}\int_{\Omega }\beta u\;dA=-\bar{\beta }\bar{I}\frac{S}{S_{0}}. \end{array}$$ (17)

Similarly,

$$\begin{array}{cc} \frac{d}{dt}\bar{I} & =\int_{\Omega }\beta I\frac{S}{S_{0}}-\gamma I+\mu (I*g-I)\;dA=\bar{I}\int_{\Omega }\beta \frac{I}{\bar{I}}\frac{S}{S_{0}}-\gamma \frac{I}{\bar{I}}+\mu (\frac{I}{\bar{I}}*g-\frac{I}{\bar{I}})\;dA\\ & =\bar{I}\int_{\Omega }\beta u\frac{S}{S_{0}}-\gamma u+\mu (u*g-u)\;dA=\bar{\beta }\bar{I}\frac{S}{S_{0}}-\bar{\gamma }\bar{I}. \end{array}$$ (18)

Finally,

$$\begin{array}{cc} \frac{\partial }{\partial t}u & =\frac{\frac{\partial }{\partial t}I}{\bar{I}}-\frac{I}{\bar{I}}\frac{\frac{\partial }{\partial t}\bar{I}}{\bar{I}}=\beta \frac{I}{\bar{I}}\frac{S}{S_{0}}-\gamma \frac{I}{\bar{I}}+\mu (\frac{I}{\bar{I}}*g-\frac{I}{\bar{I}})-\frac{I}{\bar{I}}(\bar{\beta }\frac{S}{S_{0}}-\bar{\gamma })\\ & =\left(\beta \frac{S}{S_{0}}-\gamma -\bar{\beta }\frac{S}{S_{0}}+\bar{\gamma }\right)u+\mu (u*g-u). \end{array}$$ (19)
